# Astrovirus Outbreak in an Animal Shelter Associated With Feline Vomiting

**DOI:** 10.3389/fvets.2021.628082

**Published:** 2021-02-11

**Authors:** Yanpeng Li, Emilia Gordon, Amanda Idle, Alvin Hui, Roxanne Chan, M. Alexis Seguin, Eric Delwart

**Affiliations:** ^1^Vitalant Research Institute, San Francisco, CA, United States; ^2^Department of Laboratory Medicine, University of California, San Francisco, San Francisco, CA, United States; ^3^The British Columbia Society for the Prevention of Cruelty to Animals, Vancouver, BC, Canada; ^4^IDEXX Reference Laboratories, Inc., Markham, ON, Canada; ^5^IDEXX Reference Laboratories, Inc., Westbrook, ME, United States

**Keywords:** astrovirus, feline, vomiting, metagenomic, mamastrovirus, cat

## Abstract

An outbreak of cat vomiting was observed in an animal shelter. Testing for known enteric feline pathogens did not identify a causative agent. Viral metagenomics on four mini pools of feces from cases and controls housed in the same area revealed the presence of feline astrovirus in all pools. Also found with fewer reads in one pool each were rotavirus I, carnivore bocaparvovirus 3, norovirus (NoV) GVI, and a novel dependovirus. The genome of the highly prevalent astrovirus was sequenced and classified into mamastrovirus species two, also known as feline astrovirus. Real-time RT-PCR on longitudinally acquired fecal samples from 11 sick cases showed 10 (91%) to be shedding astrovirus for as long as 19 days. Affected cats were sick for an average of 9.8 days, with a median of 2.5 days (range = 1–31 days). Unaffected control cats housed in the same areas during the outbreak showed five out of nine (56%) to also be shedding astrovirus. Feline fecal samples collected from the same animal shelter ~1 year before (*n* = 8) and after (*n* = 10) showed none to be shedding astrovirus, indicating that this virus was temporarily associated with the vomiting outbreak and is not part of the commensal virome for cats in this shelter. Together with the absence of highly prevalent known pathogens, our results support a role for feline astrovirus infection, as well as significant asymptomatic shedding, in an outbreak of contagious feline vomiting.

## Introduction

Astroviruses are non-enveloped, single-stranded, positive-sense RNA viruses with a spiked spherical shape reminiscent of a star. In 2019, the family Astroviridae included the genera *Avastrovirus* infecting birds and *Mamastrovirus* infecting mammals with three and 19 International Committee on Taxonomy of Viruses (ICTV) designated species, respectively, although many additional species have been recently proposed (1). The first astrovirus was identified in 1975 from human stool, and astrovirus infection has since been implicated as a common cause of diarrhea in children worldwide (2–4). Evidence is accumulating that recombination and cross-species transmissions may be common (5–7). More recently, astroviruses have been associated with fatal encephalitis in immune-compromised humans, as well as in cows, pigs, mink, and sheep (8–17). The role of astroviruses in enteric disorders of animals is less clear, but may occur in cattle either alone or as co-infections in bovine diarrhea (18–20). While novel astroviruses are rapidly being sequenced from many birds and mammals, evidence of their pathogenicity has been limited, restricted largely to turkeys and lambs, where disease signs range from severe to mild (21, 22). Frequent co-detection of astrovirus with other enteric viruses may contribute to increased pathogenicity. Remarkably, the capsid alone of turkey astrovirus 2 (in the absence of genomic RNA) could induce diarrhea (23).

Vomiting and diarrhea are common clinical presentations in the domestic cat (24, 25) and may be more common in shelter cats, with the incidence of diarrhea reportedly as high as 54–72% in shelter felines (26). There is little published information about the incidence of vomiting in shelter cats, but unexplained vomiting outbreaks in shelter cats in the UK have been reported (27). Many feline enteropathogens can be found in both sick and clinically healthy animals (28–32). Multiple studies have found shelter cats to be more likely than owned cats to shed various enteropathogens (33, 34). Animal shelters may be more likely to experience and identify disease outbreaks due to housing animals from various sources, constant population turnover, animal stress, and the presence of many possible exposure routes (35). Possible causes of enteric disease outbreaks include dietary or toxic causes as well as viral, bacterial, and parasitic organisms. The literature in humans suggests that outbreaks with widespread vomiting often have a viral etiology (36, 37).

Here, an outbreak of cat vomiting in an animal shelter was closely monitored, a dietary cause and common infectious agents were ruled out, and longitudinal samples were collected from both sick and healthy animals. Metagenomics analysis revealed the presence of high levels of feline astrovirus that was confirmed by real-time reverse transcription PCR (RT-PCR), providing evidence that this virus was associated with vomiting in a substantial fraction of infected cats.

## Materials and Methods

### Sample Collection and Pathogen Screening

During December 2019 and January 2020, a feline vomiting outbreak occurred in an animal shelter in British Columbia, Canada. An outbreak investigation was conducted, and a case definition was created to track all affected animals while excluding unaffected animals: cats or kittens in the shelter with vomiting (with or without diarrhea) with onset between 12/9/2019 and 1/1/2020 and no other suspected cause. In total, 12 cats met the case definition, and samples were available from 11 of these cats. Eight of these sick cats were housed individually, and four were housed in two pairs. Feces samples were available from 10 of 12 cats and vomit samples were available from seven of 12 cats meeting the case definition; in the case of pair-housed cats, pooled samples were collected. During the course of the outbreak, there were also 19 cats housed in the shelter which were not sick; fecal samples were collected from nine of these individually housed cats to use as unaffected controls. Longitudinal samples were collected where possible from both affected and control cats (cats were not kept in the shelter for the sole purpose of obtaining samples), with the total number of samples per cat ranging from 1 (cats 571 and 181) to 18 (cat pair 350/351). Fecal and vomit samples were collected into sterile plastic laboratory submission vials with a small plastic scoop or clean tongue depressor by staff wearing gloves changed between each sample. Plastic scoops and tongue depressors were single use.

Fecal samples from 10 affected cats (six individual, two pooled pairs; 10 in total) and nine control cats were submitted to IDEXX Laboratories, Inc. (Sacramento, CA, USA) for a comprehensive multi-pathogen feline diarrhea screening panel. This RT-PCR panel includes *Clostridium perfringens* alpha toxin, *C. perfringens* enterotoxin, *Campylobacter coli* and *Campylobacter jejuni, Cryptosporidium* spp., *Giardia* spp., *Salmonella* spp., *Tritrichomonas foetus, Toxoplasma gondii*, feline panleukopenia virus (FPV), and feline enteric coronavirus (FECV). There is no commercially available feline vomiting panel, but many enteropathogens that cause diarrhea may also cause vomiting. For sick cats, most samples were collected during the acute phase of illness (**Figure 2**, see number sign label). Treatments, if any, were generally initiated after sample collection.

Due to the unusual nature of this outbreak (vomiting spreading rapidly within the population, with no diarrhea in most animals), sample collection for possible further analysis commenced the day the outbreak was identified. The samples were frozen immediately and kept frozen until they were shipped on dry ice. After initial analysis of the outbreak samples, additional control samples were collected and tested by real-time RT-PCR to assess whether feline astrovirus is part of the usual virome of cats housed in the affected shelter. Eight frozen feline fecal samples from a prior outbreak research study (38) collected between November 2018 and January 2019 and 10 fecal samples from healthy shelter cats collected September 26–27, 2020 were identified for analysis.

### Sample Processing, Metagenomics Library Preparation, and Bioinformatics

The method used was identical to that previously described (38). One gram of feces was vortexed in 2 ml phosphate-buffered saline (PBS) with zirconia beads. Viral particles were then enriched by filtration through a 0.45-μm filter (Merck Millipore, MA, USA). The filtrate was digested with a mixture of nuclease enzymes prior to nucleic acid extraction using a MagMAX Viral RNA Isolation Kit (Ambion, Inc, Austin, TX, USA). Nucleic acids (both RNA and DNA) were then amplified using random RT-PCR. Briefly, reverse transcription was performed with primer with a random nonamer at the 3′ end (5′-GCCGACTAATGCGTAGTCNNNNNNNNN), followed by second-strand synthesis using Klenow Fragment DNA polymerase (New England Biolabs, MA, USA). Both complementary DNA (cDNA) and DNA were then amplified by AmpliTaq Gold DNA polymerase (Thermo Fisher Scientific, MA, USA) using a primer consisting of the fixed portion of the random nonamer containing primer (5′ GCCGACTAATGCGTAGTC). An Illumina library was then generated using the transposon-based Nextera XT Sample Preparation Kit and sequenced on the MiSeq platform (2 × 250 bases, dual barcoding; Illumina, CA, USA) with dual barcoding, as previously described (method N1) (39).

Sequence data analysis was performed as previously described (40). Adaptor and primer sequences were trimmed using the default parameters of VecScreen, which is part of the BLAST package v2.2.31. Reads were considered duplicates if base positions 5–55 were identical and one random copy was kept. Low sequencing quality tails were trimmed using Phred quality score 20 as the threshold. Bacterial reads were subtracted by mapping to the bacterial nucleotide sequences from the BLAST NT database using Bowtie2 v2.2.4 (41). The Ensemble Assembler program (v1.0) (42) was used for *de novo* assembly. Both contigs and singlets were then analyzed using BLASTx (v.2.2.7) to an in-house viral proteome database. The significant hits to viral protein (*E* scores < 0.001) were then aligned to the BLAST NR universal proteome database using DIAMOND v0.9.15.116 and retained only if the most significant alignment (i.e., the lowest *E* score) was to a viral protein sequence.

Viral reads and contigs were aligned to reference viral genomes to generate full/partial genome sequences by the Geneious R11 program (43). Nucleotide sequences were first translated into amino acids and aligned using ClustalW. Phylogenetic trees were inferenced using maximum likelihood method with MEGA 7.0 (44). The model test module of MEGA 7.0 was used to determine the best substitution model. Phylogenetic trees based on nucleotide sequences were generated using the bootstrap method (1,000 times) under a GTR+I+G model.

### Real-Time PCR Assay

RNA was extracted using Qiagen Viral Mini Kit (Qiagen, Germany) and reverse transcribed to cDNA using SuperScript III first-strand synthesis kit (Thermo Fisher). The primers and probe were designed based on the assembled astrovirus genome: forward primer 5′-GGATTGGGCATGGTTTAGAG-3′, reverse primer 5′-ACGCATCCAGGTAGTCTTC-3′, probe 5′-FAM-AGGATAGGCAGGTGTTGATTCAGTGTTT-BHQ-3′. The real-time PCR conditions are 95°C for 3 min, 45 cycles of 95°C for 10 s, and 60°C for 30 s (two-step).

### Data Availability

The short reads sequencing data from the four mini pools of fecal samples are available at the NCBI Sequence Read Archive (SRA) under the BioProject number PRJNA565775 (SAMN16266519-16266522). GenBank accession number for feline astrovirus BC SPCA is MW164633.

## Results

### Epidemic and Clinical Data

A feline vomiting outbreak was identified in an animal shelter in British Columbia, Canada, from December 2019 to January 2020. Of the 31 cats housed in the shelter during the outbreak period, 12 cats met the case definition (see *Materials and Methods*). Fecal or vomit samples from 11 of the 12 sick cats and 9 of 19 unaffected cats during the outbreak period were available for further study, as well as samples from 18 other cats from the same shelter collected approximately a year before and after the outbreak period.

**Figure 1:**
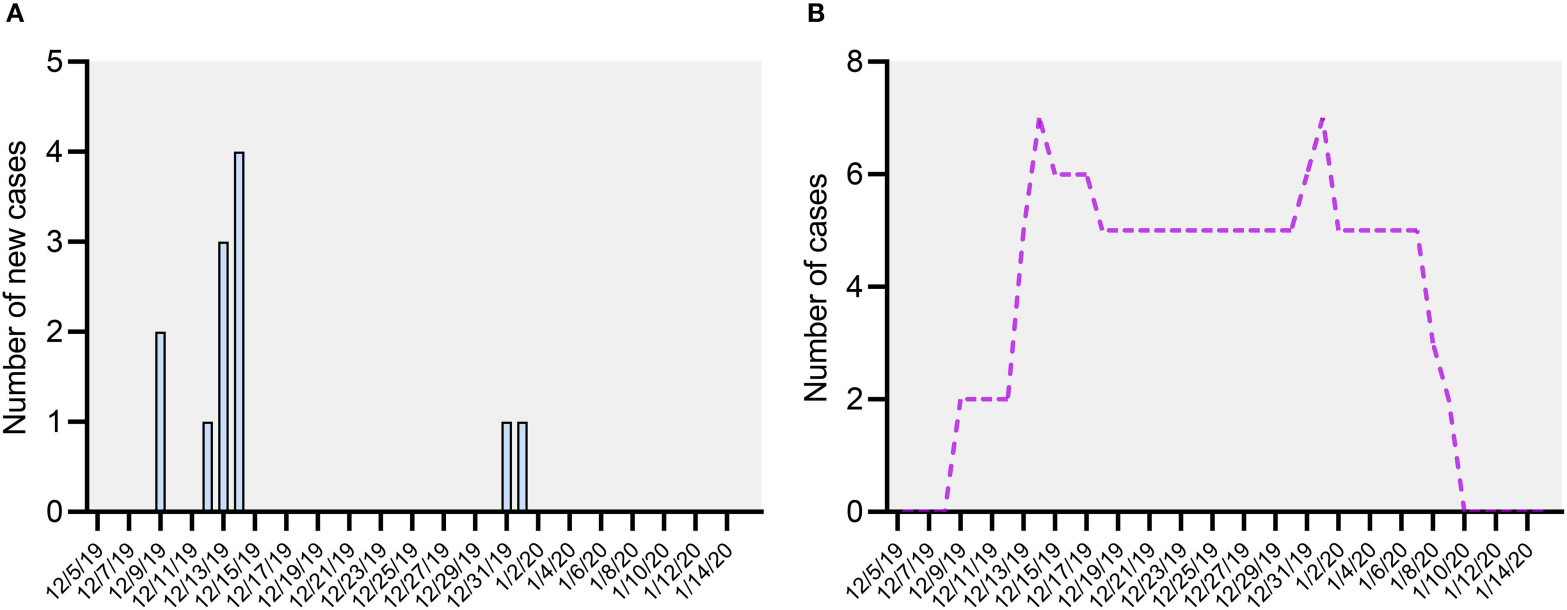
**(A)** Day of illness onset for cats meeting the case definition, December 2019–January 2020. **(B)** Number of active cases meeting the case definition, December 2019–January 2020.

The outbreak was initially identified on December 13, 2019, when six cats in two different rooms in the shelter were noted to have vomited over the previous few days. One cat also had diarrhea for a single day. On December 14, four more cats began vomiting, including two cats in a third room; at that point, a total of 10 of 17 cats housed in the shelter were affected (Figure 1A, outbreak). An outbreak investigation was launched and dietary, environmental, and toxic causes ruled out. The first cases were two cats (598 and 277) in two different rooms which both began vomiting on December 9, with more cases appearing rapidly in both rooms. This likely indicates that the true index case was never identified; perhaps there was a cat present prior to December 9 which was shedding without clinical signs.

Except for co-housed pairs 541/542 and 350/351, none of the affected cats had any direct contact, indicating an indirect transmission route for most cases. Most affected cats were housed in enriched two to three-compartment individual cages of 11–16 ft^2^ total, but several were housed in small rooms. Control measures were implemented on December 14, including cessation of all cat movement, use of personal protective equipment changed between each cage (gown, gloves, caps, and shoe covers) in all cat housing areas, and enhanced sanitation measures using accelerated hydrogen peroxide. This disinfectant has good efficacy against bacteria and viruses, including non-enveloped viruses (45).

After control measures were initiated, there were no new clinical cases until December 31 (cat 181), followed by another case (cat 235) on January 1. Because five cats from the first wave of illness experienced prolonged clinical signs, there were still active cases in the shelter at the time of the onset of these cases (Figure 1B, active case). Both of these cats were only mildly ill, with an illness duration of 1–2 days. After January 1, there were no further new cases.

Due to most cases likely resulting from indirect transmission, it was not possible to determine the exact exposure dates for many cats, nor whether control animals were actually exposed. The minimum incubation period in this outbreak was determined to be 3 days based on three cats (cats 541, 542, and 496) which entered the shelter on December 11 prior to the initiation of control measures and became sick on December 14, as well as one cat (cat 285) which entered the shelter on December 29 and got sick on January 1. It was not possible to determine a maximum incubation period with the data available. The clinical attack rate before the control measures were implemented was 59% (10 of 17) and after control measures was 17% (2 of 12).

Vomiting was observed in 100% (12 of 12) of the cases and diarrhea was observed in only one cat (cat 277). Inappetence was noted in association with vomiting in four cats; this always coincided with and/or followed vomiting and was never the first clinical sign observed. The mean duration of illness was 9.8 days and the median was 2.5 days, with range of 1–31 days (Supplementary Table 1, clinical signs). Cats tended to either recover rapidly (seven cats were sick for 1–4 days) or experience prolonged illness (five cats had a total illness duration of 25–31 days). While some of the cats in the first wave of the outbreak recovered rapidly, all five of the cats with prolonged illness and all cats requiring outside veterinary care became sick during the first wave of the outbreak (before control measures were implemented on December 14; the two cases with onset after the initiation of control measures were both mild and self-limiting). Within the total illness duration, cats experienced 1–13 actual episodes of illness, and in prolonged cases, vomiting became sporadic before ceasing.

### PCR Pathogen Screening

**Figure 2:**
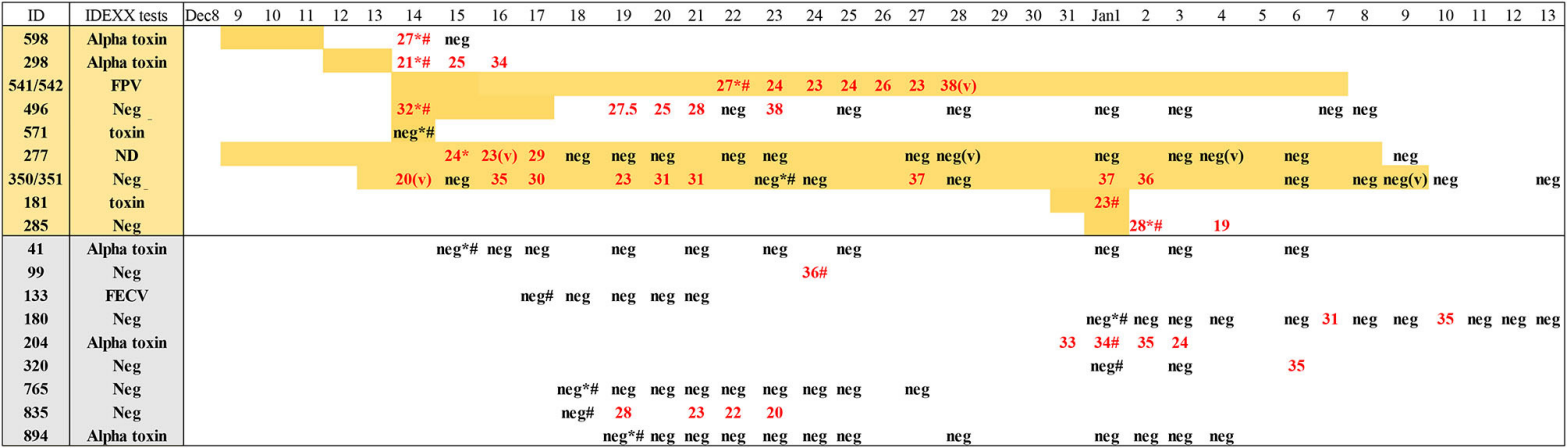
Real-time RT-PCR for feline astrovirus of samples from affected and control animals showing *C*_t_ numbers and results of IDEXX testing for feline diarrhea pathogens. Samples of all feces, unless labeled (*v*) for vomit; *numbers in red* indicate *C*_t_ for astrovirus RT-PCR. Negative for *C*_t_ > 40. *Number symbol*, sample used for IDEXX testing. *Asterisk*, sample used for metagenomic next-generation sequencing (mNGS). Yellow highlight indicate days of vomiting. *ND*, not done.

Eighteen fecal samples representing 20 cats (16 individual and four pair-housed) were available for testing with a commercially available feline multi-pathogen diarrhea real-time PCR panel (Figure 2, samples analyzed labeled with a number sign). Where multiple longitudinal fecal samples were available, the first sample was analyzed. Of the clinically affected cats, three were weakly positive for *C. perfringens* alpha toxin gene, two were weakly positive for FPV (one sample was a pool of feces from a pair of kittens, no. 541/542), and one was positive for FECV (Figure 2, IDEXX tests column). The *C. perfringens* alpha toxin gene can be detected at low levels in both healthy and diarrheic cats and is not considered a primary cause of enteric illness in cats (32, 46). Feline panleukopenia can also be detected in feces by PCR after modified live vaccination or field infection (47); neither FECV nor FPV was considered a likely cause for this outbreak based on their expected clinical patterns and the detection of each in only one (FECV) or two (FPV) affected cats. The detection rates for the *C. perfringens* alpha toxin and FECV were similar for both the affected and control cats in this study (Figure 2, IDEXX test column). Screening for common infectious agents using the comprehensive feline diarrhea PCR panel therefore did not yield a potential causative agent for the outbreak.

### Metagenomics

Metagenomics analysis was performed on 12 fecal samples from 14 animals (cage mate pairs were analyzed as a single mixed sample) including 10 vomiting and four unaffected cats. The samples from the disease cases were collected as early as possible during the course of their disease, except for the 350/351 pair which was analyzed 10 days after onset (Figure 2, samples analyzed labeled with an asterisk). Four pools of fecal samples from three cats each (co-housed pairs counted as one) were generated, from which viral particle-associated nucleic acids were enriched (Table 1). Following nucleic acid extractions, both RNA and DNA were converted into an Illumina MiSeq library using the transposon-based Illumina kit Nextera XT (see *Materials and Methods*). Viral sequences were identified using *in silico* translated protein sequence similarity to all eukaryotic viruses whose genomes are available in GenBank. In each of the four pools was found a unique mammalian virus at a low concentration based on the small number of recovered sequence reads as well as feline astrovirus with higher numbers of viral reads (Table 1). These virus detections consisted of 42 reads of rotavirus I, two reads of norovirus (NoV) GVI, six reads of feline bocavirus, and 112 reads of feline dependovirus in pools 1 through 4, respectively. In the same four pools, an average of 220,858 reads of feline astrovirus was detected (range = 6,741–471,237). Because feline astrovirus was detected in every pool, each of which included a sample from one to three sick cats, subsequent studies focused on the association of feline astrovirus and this vomiting outbreak. The other four detected viruses were considered incidental infection unrelated to this outbreak.

**Table 1:** Virus metagenomics analysis of four pools of fecal samples.

**Pool no**.	**Cat ID**	**SRA accession**	**Total reads**	**Astrovirus reads**	**Other viruses**	**Other virus reads**
1	(***350/351***), ***277***, 180	SAMN16266519	778,338	5,8667	Rotavirus I	42
2	***496***, ***571**,* 041	SAMN16266520	1,238,996	6,761	NoV GVI	2
3	(***541/542***), ***298**, **285***	SAMN16266521	1,374,874	346,769	Feline bocavirus	6
4	***598***, 894, 765	SAMN16266522	1,260,146	471,237	Dependovirus	112

*Cat IDs in bold and italic indicate sick cats. Bracketed pairs were co-housed in the same cage*.

### Feline Astrovirus Genome

**Figure 3:**
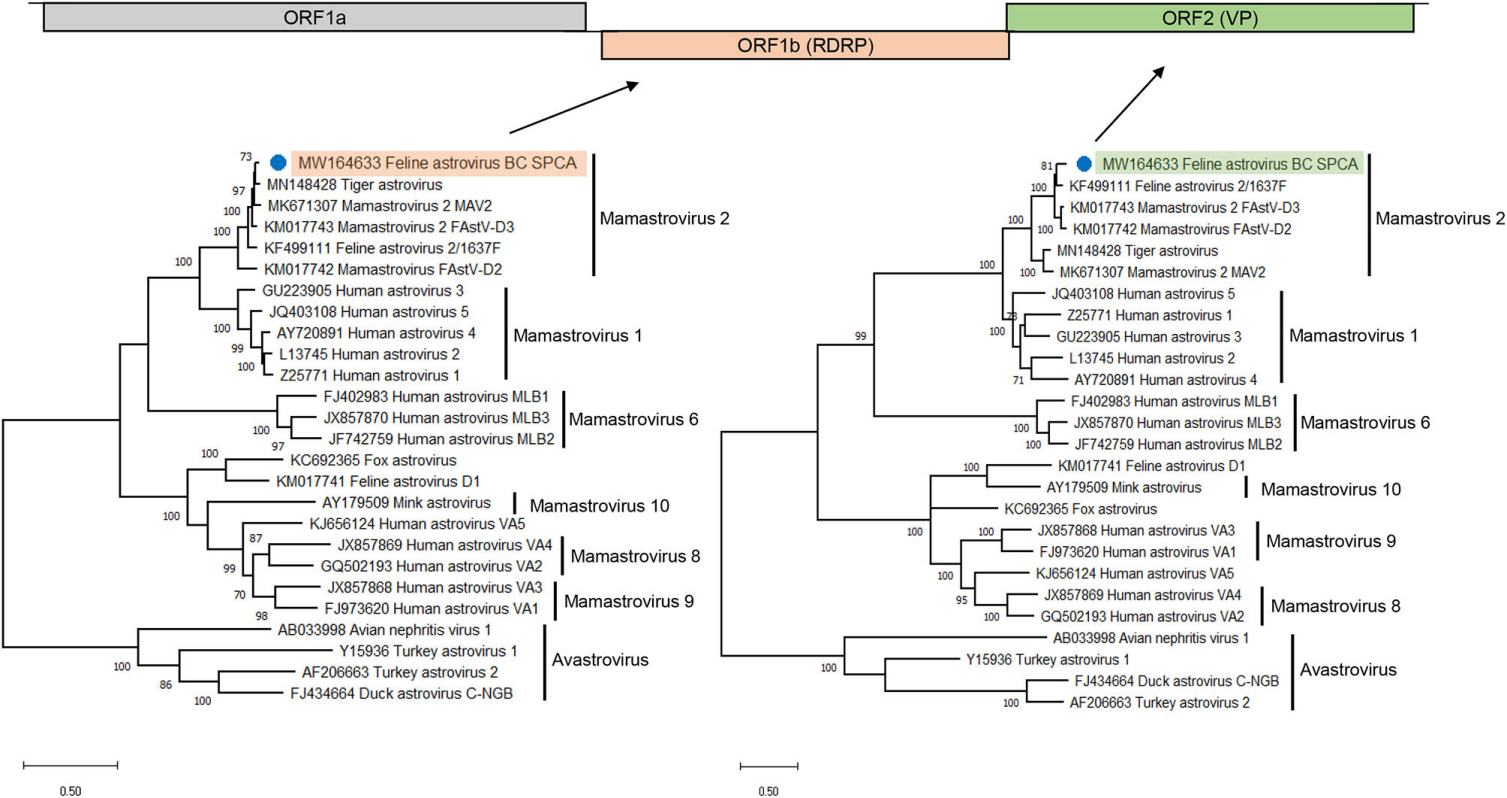
Phylogenetic analysis of RdRp **(left)** and the capsid region **(right)** of feline astrovirus in a shelter outbreak. Phylogenetic trees were inferred by the maximum likelihood method based on nucleotide sequences. Related astrovirus from mamastrovirus were included for analysis. Astroviruses were used as the outgroup; the astrovirus detected in this study was highlighted. Branch length are scaled to the number of nucleotide substitutions per site.

The near complete astrovirus genome was assembled by reference mapping reads against feline astrovirus, resulting in a contig that included both major open reading frames (ORFs) typical of astroviruses (GenBank accession number MW164633). When these proteins were phylogenetically analyzed, they fell squarely into the carnivore astrovirus 2 species (mamastrovirus species 2), in which have been reported the large majority of astroviruses from cats as well as astroviruses from Siberian tigers and cheetahs (48, 49) (Figure 3).

### Real-Time RT-PCR Astrovirus Test

A real-time RT-PCR was then designed and the presence and concentration of astrovirus RNA measured in each available fecal sample from 11 cases (four analyzed as two pairs) and nine controls. The results showed that all but one out of 11 sick cats were positive for astrovirus RNA (Figure 2). Astrovirus shedding lasted as long as 20 days while an animal was sick before turning negative during the last few days of its disease (pair 350/351). For cat 277, shedding was detected at the earliest time point available, 7 days after sign onset for 3 days followed by 23 RT-PCR-negative days despite continued vomiting. For the other affected cat samples, disease signs were shorter in duration and the sampling limited to a single (cats 181 and 571) or only a few days. The only vomiting cat in which the astrovirus was not detected (cat 571) was sampled at only a single time point on the same day that the vomiting started before the cat was returned to its owner the following day. As a comparison group, samples were also collected from nine healthy cats housed in the same shelter rooms as affected cats during the outbreak and similarly tested by real-time RT-PCR. Five of these nine healthy cats showed the presence of astrovirus RNA (Figure 2). In two healthy cats, astrovirus RNA was detected over a period of at least 4–5 days.

### Prevalence of Feline Astrovirus in Shelter Over Time

In order to determine whether feline astrovirus was a chronic presence in either this shelter or in the feline population served by this shelter, we tested other cats by real-time RT-PCR. Fecal samples from cats from the same shelter collected either a year before (*n* = 8) and after (*n* = 10) this outbreak all tested negative for the astrovirus. Highly prevalent astrovirus infections were therefore a singular, rather than a frequent, occurrence for this shelter and corresponded in time to an outbreak of feline vomiting.

## Discussion

The earliest report of astrovirus infection in a diarrheic kitten was made with electron microscopy (50). The first feline astrovirus sequence information were of capsid proteins (51, 52) and the first complete genome reported in 2013 (KF499111.1) (53). Feline astroviruses consist of members of the mamastrovirus 2 species, the most closely related species to the eight genotypes of classic human astroviruses comprising mamastrovirus 1 species. This close relationship reflects a recent common ancestry or cross-species transmission (54). Members of mamastrovirus 2 have also been reported in Siberian tigers in a Chinese zoo (48), and a close viral relative was associated with an outbreak of diarrhea in cheetahs (49). Evidence for recombination with one of the numerous porcine astroviruses was also described (55). A second highly divergent feline astrovirus (KM017741) was also sequenced in 2014 (56) and close relatives later detected in another two cats using consensus RT-PCR (57). This same study also detected an (avian) avastrovirus and a novel mamastrovirus whose closest, yet still distant, relative was from a Chinese bat (57). It was postulated that these astrovirus reads may be derived from consumed animals in these cats' diet, such as birds and bats, with these preys' viruses passively transiting through the gut (57). The more common members of the mamastrovirus 2 species have been reported at high frequency in numerous feline studies (55–58), although not generally significantly associated with diarrhea (59–61). A single study of cats in China showed an association between diarrhea and astrovirus detection (62).

To our knowledge this is the first publication describing a disease outbreak in domestic cats characterized by vomiting and a near-complete absence of diarrhea. We show that feline vomiting occurred co-incidentally with the detection of astrovirus infection in nearly all the affected cats and in half of the healthy cats housed in the same shelter during the outbreak period. None of the cats tested a year before and after the vomiting outbreak showed astrovirus shedding. Multiple cats tested positive for the *C. perfringens* alpha toxin, and smaller numbers tested positive for FPV and FECV in both the affected and control groups at similar rates; none of these pathogens were clinically consistent with being the causative agent for the outbreak as a whole. Despite extensive pathogen screening including viral metagenomics, no other highly prevalent, credible cause was found. Astrovirus was detected in both feces and vomit, suggesting that viral replication occurs in both the upper and lower gastrointestinal tracts. As most affected cats (10 of 11) and about half of the healthy cats (5 of 9) shed astrovirus, this difference was not significant (*p* > 0.05, chi-square test), reflecting a large fraction of asymptomatic infections as is common for numerous enteric viral infections (63–65).

Interestingly, during the first wave of the outbreak (defined as cats with illness onset on or prior to December 14), all clinically unaffected cats tested were negative for astrovirus. Only cats potentially exposed after the control measures were implemented on December 14 were found to shed astrovirus despite having no clinical signs. Although the sample size is very small, the cats which became sick after the control measures were implemented had milder illness than the cats in the first wave. Therefore, another consideration may be that the size of the inoculum is a determinant in the development of clinical signs. General viral contamination, and therefore the size of the viral dose in any exposed animal, would be expected to be much lower when control measures are in place.

This outbreak was unusual because vomiting was the predominant clinical sign; most enteric illnesses in shelter animals involve both vomiting and diarrhea, and diarrhea is much more widely studied. Another unusual feature was the importance of indirect routes of transmission and the persistence of sporadic, low-level transmission for several weeks after robust control measures were implemented. These were also seen in a vomiting and diarrhea outbreak associated with a novel parvovirus, named fechavirus, we recently described (38). These features, along with the rapid spread and lack of identification of other potential causative agents on screening tests, are most consistent with a non-enveloped enteric virus as the causative agent. This highlights the importance of good infection control and biosecurity measures that include consideration of non-enveloped viruses in both clinical and non-clinical animals in shelters, veterinary hospitals, and catteries.

In this study, we describe a feline vomiting outbreak in an animal shelter and characterize the virome and shedding of feline astrovirus in both affected and control cats. The vast majority of sick cats and half of the control cats during the outbreak period were shedding feline astrovirus, some for several weeks. Samples from cats in the same shelter taken well before and after this outbreak showed no feline astrovirus, suggesting that it is not a part of the usual virome for cats in this shelter. Testing fecal samples using a commercial feline diarrhea panel did not reveal any pathogenic candidate. Our results therefore support a pathogenic role for feline astrovirus infection in an outbreak of contagious feline vomiting.

## Data Availability Statement

The short reads sequencing data from the four mini-pools of fecal samples are available at NCBI Sequence Read Archive (SRA) under the BioProject number PRJNA565775 (SAMN16266519-16266522) and GenBank accession number MW164633.

## Author Contributions

YL, EG, and ED conceptualized the study. YL, EG, AI, AH, MS, and RC curated the data. YL, EG, and ED contributed to formal analysis. YL and EG designed the methodology. ED and EG supervised the study. YL, EG, and ED wrote the original draft. YL, EG, ED, MS, and RC reviewed and edited the manuscript. All authors contributed to the article and approved the submitted version.

## Conflict of Interest

RC and MS are employed by company IDEXX Reference Laboratories, Inc. The remaining authors declare that the research was conducted in the absence of any commercial or financial relationships that could be construed as a potential conflict of interest.
